# Circular RNA circ_0004488 Increases Cervical Cancer Paclitaxel Resistance via the miR-136/MEX3C Signaling Pathway

**DOI:** 10.1155/2022/5435333

**Published:** 2022-11-17

**Authors:** Hanjie Yi, Yongqing Han, Qin Li, Xia Wang, Le Xiong, Shanfeng Li

**Affiliations:** ^1^Department of Oncology, The Second Affiliated Hospital of Nanchang University, Nanchang 330000, Jiangxi, China; ^2^Department of Oncology, ShangRao People's Hospital, Shangrao 334000, Jiangxi, China; ^3^Department of Infection Management, The Second Affiliated Hospital of Nanchang University, Nanchang 330000, Jiangxi, China

## Abstract

Circular RNAs have been proven to play a pivotal role in cervical cancer development, progression, and treatment resistance. However, it is unclear how these RNAs influence chemoresistance in cervical cancer, particularly cancer stem cell (CSC)-like properties. In this study, we found that circRNA circ_0004488 was highly expressed in CSC-enriched subsets of cervical cancer cell lines. The expression of circ_0004488 was upregulated in cervical cancer cells that were resistant to paclitaxel. When circ_0004488 expression was high, the prognosis was poor. Specifically, we discovered that knocking down circ_0004488 greatly decreased the development of cervical cancer cells in vivo by decreasing cell proliferation, invasion, and sphere formation. By blocking cir_0004488, cervical cancer cells become more sensitive to paclitaxel. In cervical cancer cells, circ_0004488 acted as a microRNA-136 (miR-136) sponge, increasing the expression of MEX3C (a direct target gene of miR-136) using dual-luciferase reporter assays. Moreover, MEX3C downregulation significantly reduced cell proliferation, invasion, sphere formation, and paclitaxel resistance. In conclusion, circ_0004488 was shown to induce CSC-like features and paclitaxel resistance through the miR-136/MEX3C axis. Therefore, circ_0004488 might be a good therapeutic target for treating cervical cancer.

## 1. Introduction

The self-renewal and differentiation capabilities of cancer stem cells (CSCs) are a major therapeutic challenge [1] since they are the cells responsible for tumorigenesis, metastasis, and recurrence following chemotherapy. Higher resistance to cancer treatment has been linked to the presence of key regulators of strain self-renewal, including octamer-binding transcription factor 4 (OCT4), sex-determining region Y-box 2 (SOX2), and the Nanog homeobox (NANOG) [2, 3]. Cervical cancer ranks as the fourth most prevalent malignancy in females [3]. Patients diagnosed with cervical cancer now have a better prognosis because of improvements in both prevention and treatment. Unfortunately, advanced cervical cancer is incurable owing to relapses, metastases, or a lack of suitable therapeutic options [4]. Cervical cancer has been treated with platinum and paclitaxel (PTX) [4]. However, PTX resistance is a huge obstacle to effective cervical cancer therapy [5]. Therefore, novel therapies are required to improve therapeutic outcomes in cervical cancer patients.

In cervical CSCs, PTX resistance has been demonstrated [6]. Cervical CSCs have been reported, and they are treatment-resistant [6]. Cervical CSCs were enriched using sphere formation assays, and sphere-forming cervical CSCs were more invasive and resistant to chemotherapeutics than control cells [7]. MEX3C is a protein with two K homology (KH) RNA-binding domains that has a role in energy metabolism, immune response, and cancer growth [8]. MEX3C has previously been demonstrated to activate JNK signaling, which promotes bladder carcinogenesis [8]. Activation of the JNK pathway is important for PTX resistance in ovarian cancer [9]. Although MEX3C has been implicated in cervical CSCs and PTX resistance, its influence and mechanism in these processes need to be elucidated.

MicroRNA (miRNA) is a kind of noncoding RNA that controls protein synthesis and post-transcriptional expression [10]. MiRNAs bind to the 3′-untranslated region (3′-UTR) of the mRNA they target, inhibiting transcription [10]. MiR-136 inhibits the proliferation, invasion, migration, cell cycle progression, and radiation resistance of cervical cancer cells [11–13]. By targeting Notch3, MiR-136 inhibits ovarian cancer stem cell activity and increases PTX antitumor efficacy in ovarian cancer cells [14]. MiR-136 inhibited ovarian cancer cell proliferation, migration, and invasion while increasing cell death and susceptibility to PTX [15]. However, little is known regarding the effect of miR-136 on PTX resistance in cervical cancer.

Noncoding RNAs with a circular loop structure, known as circular RNAs (circRNAs), regulate gene expression via complex mechanisms [16]. Many circRNAs play crucial biological roles as miRNAs or protein sponges [16]. CircRNAs are well recognized for regulating cervical cancer development and drug resistance [17]. A recent research study found that circRNA hsa_circ_0004488 was more highly expressed in cervical cancer than in comparable nearby normal tissues [18]. However, to the best of our knowledge, the role and regulatory mechanism by which circ_0004488 mediates PTX resistance, as well as cervical CSCs, are largely unresolved.

In this research study, we observed that circ_0004488 enhanced CSC-like characteristics and PTX resistance via the miR-136/MEX3C axis. Therefore, inhibiting circ_0004488 may represent a potential therapeutic method for the treatment of cervical cancer.

## 2. Materials and Methods

### 2.1. Ethics Statement

This study was conducted using human cervical cancer tissue samples by following the ethical standards of the Helsinki Declaration of 1975 and was approved by the Ethics Committee of the Second Affiliated Hospital of Nanchang University. Written informed consent was obtained from all patients. Moreover, all experimental protocols in the animal studies were performed in compliance with the institutional ethical standards, the ARRIVE guidelines (https://arriveguidelines.org), and all relevant guidelines and regulations. All animal studies were conducted in compliance with the China Council on Animal Care and Use Guidelines and approved by the Animal Experimentation Ethics Committee of the Second Affiliated Hospital of Nanchang University.

### 2.2. Sphere Formation Assays

Single cells were plated in 96-well ultralow adherence plates from Corning (Lowell, MA, USA) at a density of 500 cells per well and grown in a serum-free DMEM medium supplemented with 20 ng/ml epidermal growth factor (R&D Systems, Minneapolis, MN, USA), 20 ng/ml basic fibroblast growth factor (R&D Systems), and B-27 supplement from Thermo Fisher Scientific, Waltham, MA, USA. The amount and size of the spheres produced were measured using a microscope after 14 days.

### 2.3. Cell Lines

HeLa, C33A, and SiHa cervical cancer cell lines and a normal Ect1/E6E7 cell line were purchased from the American Type Culture Collection (ATCC, Manassas, VA, USA) and grown in DMEM (Sigma-Aldrich) media supplemented with 10% fetal bovine serum (FBS, Thermo Fisher Scientific). Paclitaxel-resistant SiHa (SiHa/PTX) cells were developed by exposing SiHa cells to gradually increasing amounts of paclitaxel (Sigma-Aldrich) [19]. All cells were incubated at 37°C in a humidified incubator containing 5% CO_2_.

### 2.4. Cancer and Normal Tissues

The patients who received cervical cancer resection at the Second Affiliated Hospital of Nanchang University provided human cervical cancer tissues and matched normal tissues. Each patient provided written and informed permission. Preoperative chemotherapy or radiation therapy was not given to any of the patients. This research was approved by the Institute Research Ethics Committee of the Second Affiliated Hospital of Nanchang University. Snap-frozen tissue samples were kept at −80°C after being frozen in liquid nitrogen.

### 2.5. qRT-PCR Assays

The RNAiso Plus kit was used to extract the total RNA from cervical cancer, neighboring normal liver tissues, and cells (Takara, Japan). RNA was extracted from nuclear and cytoplasmic fractions using nuclear and cytoplasmic extraction kits from Thermo Fisher Scientific. mRNA and circRNA were reverse transcribed using Prime Script RT Master Mix (TaKaRa, Japan). SYBR Green was used in real-time PCR testing (qRT-PCR) on cDNA (TaKaRa). Using the miRNA qRT-PCR Starter kit, miRNA expression was evaluated (Ribobio, China). Internal controls were performed using GAPDH and U6. The PCR primers used for the detection of circ_0004488 were designed using the CircInteractome database (https://circinteractome.nia.nih.gov/index.html). These primers span the back-splicing junction of circ_0004488. The following primers were used: Hsa_circ_0004488 forward (F): 5′-ATGGTCAGAAGTGTGGCTGC-3′; hsa_circ_0004488 reverse (R): 5′-CGGAGCTGAAGAGCTCAATGT-3′; PRPSAP2 F: 5′- TGTGCAAAGCTGGTCTAACTC-3′ and PRPSAP2 R: 5′- GGCGCTCAGCAAAAGACTG-3′; SOX2 F: 5′-GCCGAGTGGAAACTTTTGTCG-3′ and SOX2 R: 5′- GGCAGCGTGTACTTATCCTTCT-3′; NANOG F: 5′-TTTGTGGGCCTGAAGAAAACT-3′ and NANOG R: 5′-AGGGCTGTCCTGAATAAGCAG-3′; OCT4 F: 5′-CTGGGTTGATCCTCGGACCT-3′ and OCT4 R: 5′-CCATCGGAGTTGCTCTCCA-3′; KLF4 F: 5′-CCCACATGAAGCGACTTCCC-3′ and KLF4 R: 5′-CAGGTCCAGGAGATCGTTGAA-3′; CD133 F: 5′-AGTCGGAAACTGGCAGATAGC-3′; and CD133 R: 5′-GGTAGTGTTGTACTGGGCCAAT-3′; CD44 F: 5′-CTGCCGCTTTGCAGGTGTA-3′ and CD44 R: 5′-CATTGTGGGCAAGGTGCTATT-3′; GAPDH F: 5′-AATCCCATCACCATCTTC-3′ and GAPDH R: 5′-AGGCTGTTGTCATACTTC-3′; U6 F: 5′-GCTTCGGCAGCACATATACTAAAAT-3′ and U6 R: 5′-CGCTTCACGAATTTGCGTGTCAT-3′.

### 2.6. RNA Separation from Nuclear and Cytoplasmic Locations

The PARIS kit (Life Technologies) was used to extract cytoplasmic and nuclear RNAs. The qRT-PCR test was used to determine the quantity of circ_0004488, with GADPH and U6 (F: CTCGCTTCGGCAGCACA and R: AACGCTTCACGAATTTGCGT) serving as reference genes for cytoplasmic and nuclear RNA, respectively.

### 2.7. Plasmids, siRNAs, and Transfection

GenePharma Biotech (Shanghai, China) created the siRNAs directed against circ_0004488 or MEX3C as well as the negative control siRNA. The siRNAs were transfected into cervical cancer cells using Lipofectamine 3000 (Thermo Fisher Scientific). According to the manufacturer's instructions, human circ_0004488 was amplified and introduced into an overexpression vector (Geneseed, China) before being transfected with Lipofectamine 3000. Following stable transfection, cervical cancer cells were selected for two weeks with 1 *μ*g/ml puromycin. Ribobio (China) supplied the miR-136 mimics, inhibitors, and their respective controls.

### 2.8. Cell Counting Kit-8 (CCK-8) Assays

In 96-well plates with a growth medium, cervical cancer cells were cultured. Three days after transfection, a CCK-8 solution (Dojindo, Japan) was added, and absorbance was measured at 450 nm using a microplate reader (Bio-Rad, Hercules, CA, USA).

### 2.9. Transwell Invasion Assays

Transwell chambers were covered with Matrigel matrix (Corning, NY, USA). Cervical cancer cells were pipetted into chambers containing 0.5 ml of FBS-free DMEM medium suspension solution, with the appropriate complete medium added to the bottom chamber. The upper side of the membrane was washed away 24 hours later, leaving the bottom of the membrane with invading cells. The cells were then stained for 15 minutes at room temperature with crystal violet. Finally, cell counts were determined using a microscope at 10 randomly selected sites on each membrane.

### 2.10. Cell Survival Assays

In 96-well tissue culture plates, cervical cancer cells were planted. Following 24 hours of culture, the wells were supplied with a paclitaxel-containing growth medium. Wells containing drug-free growing medium were used as controls. Cell viability was subsequently assessed using CCK-8 assays after the plates had been cultured for 48 hours.

### 2.11. RNA Pull-Down and RNA Immunoprecipitation Assays

RiboBio (China) created a biotin-labeled version of the circ_0004488 probe. Cervical cancer cells were fixed for 10 minutes in 1 percent formaldehyde (Sigma-Aldrich) before being lysed and sonicated. Following centrifugation, 20 *μ*l of the supernatant was kept as input, while the remainder was incubated overnight with a biotin-labeled circ_0004488 probe-streptavidin M-280 bead combination (Thermo Fisher Scientific). To reverse the formaldehyde crosslinking, the beads-RNA combination was washed and treated with lysis solution and proteinase K (Sigma-Aldrich). Finally, the TRIzol reagent (Thermo Fisher Scientific) was added to the mixture for RNA extraction. The Magna RIP kit was used for the RNA immunoprecipitation experiment (Millipore, Billerica, MA, USA). RIP buffer containing IgG-antibody or Ago2-labeled magnetic beads was utilized, and the immunoprecipitated RNAs were then produced using the TRIzol reagent (Thermo Fisher Scientific).

### 2.12. Luciferase Activity Assays

The wild-type (WT) and mutant (MUT) circ_0004488 segments, along with the 3′-UTRs of wide-type and mutant MEX3C containing miR-136 binding sites, were inserted into the pGL3 vector (Promega, Madison, WI, USA) and received from RiboBio (China). After overnight growth, these reporter vectors were cotransfected into cells using Lipofectamine 3000 and a miR-136 mimic or inhibitor. Using a dual-luciferase assay kit (Promega), luciferase activity was evaluated 48 hours after transfection, and firefly luciferase activity was adjusted relative to Renilla luciferase activity for comparison.

### 2.13. Western Blot Analysis

For western blot analysis, RIPA buffer (CWBIO, Beijing, China) was employed to generate cell lysates. Using a bicinchoninic acid protein assay kit, protein concentrations were measured (Beyotime Biotechnology, China). Using SDS-PAGE, 30 *μ*g of each protein sample was separated and then transferred to PVDF membranes (Millipore). The membranes were then incubated overnight at 4°C with the primary antibody (anti-MEX3C from Abcam or anti-*β*-actin from Cell Signaling Technology). The next day, membranes were incubated with HRP-conjugated secondary antibodies at room temperature for one hour. Millipore chemiluminescence detection reagents were used to identify signals. In this study, *β*-actin was utilized as a loading control.

### 2.14. Xenograft Models

All animal research studies were approved by the Animal Experimentation Ethics Committee of the Second Affiliated Hospital of Nanchang University. Circ_0004488 was overexpressed in HeLa cells (1 × 10^6^) before being subcutaneously delivered to 4-week-old male BALB/c nude mice (Beijing Vital River Laboratory Animal Technology, China). Tumor volume was calculated using the formula volume = 0.5 × length × width^2^. Tumors were weighed after animals were sacrificed.

### 2.15. Statistical Analysis

Group differences were evaluated using the Student's *t*-test, and statistical analysis was conducted using SPSS 20.0 (SPSS, Chicago, USA). Statistics were deemed significant at *P* < 0.05.

## 3. Results

### 3.1. Circ_0004488 Levels Were Increased in CSC-Enriched Subpopulations of Cervical Cancer Cells

**Figure 1:**
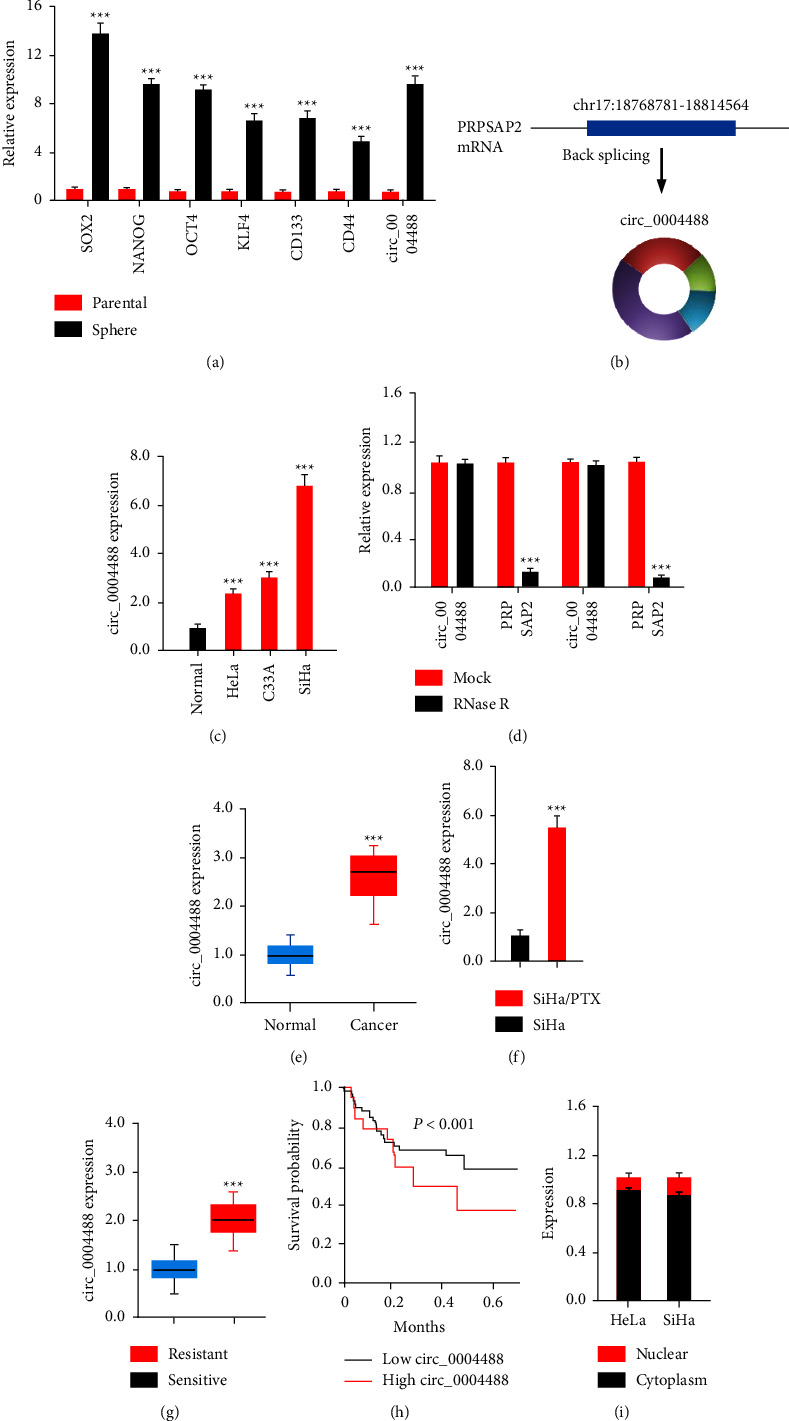
Increased circ_0004488 levels in CSC-enriched populations of cervical cancer cells. (a) QRT-PCR analysis showed that CSC-enriched spheres had a higher concentration of CSC markers compared to parental cell lines. (b) PRPSAP2 exons are back-spliced to create circ_0004488. (c) Expression of circ_0004488 was examined in cervical cancer cell lines and a normal cell line using quantitative real-time polymerase chain reaction. (d) Expression of circ_0004488 and PRPSAP2 mRNA was measured in cervical cancer cells treated with or without RNase R by quantitative real-time polymerase chain reaction. (e) Circ_0004488 expression in human cervical cancer and adjacent normal tissues. (f) Circ_0004488 expression in either paclitaxel-sensitive or -resistant SiHa cells. (g) Circ_0004488 tissue levels in paclitaxel-resistant and -sensitive cervical carcinoma. (h) A comparison of the prognosis for individuals with cervical cancer who express high levels of circ_0004488 with those who express low levels of this gene is shown in the panel. (i) Circ_0004488 was mostly located in the cytoplasm of cervical cancer cells. ^*∗∗∗*^*P* < 0.001.

Beginning with the HeLa cell line, sphere formation experiments were performed to establish a CSC-enriched population of spheres (Figure 1(a)). As expected, CSC markers such as CD133, CD44, KLF4, NANOG, SOX2, and OCT4 were much greater in CSC-enriched spheres than in parental cell lines (Figure 1(a)). The expression of circ_0004488, a circRNA generated from the PRPSAP2 gene, was much higher in the spheres than in the parental HeLa cells (Figure 1(b)), indicating that a high expression of circ_0004488 may be involved in the acquisition of CSC-like characteristics in cervical cancer cells. Three cervical cancer cell lines expressed more circ_0004488 than normal cells, as indicated by qRT-PCR assays (Figure 1(c)). CircRNAs are noncoding RNAs with a closed circular structure that are more stable than linear mRNAs [16]. CircRNAs are more stable than linear mRNAs. To determine if circ_0004488 mRNA is more stable than linear PRPSAP2 mRNA, we examined the expression of circ_0004488 and PRPSAP2 in HeLa cells after RNase R treatment. Even though the linear RPSAP2 mRNA was degraded by RNase R digestion, the amount of circ_0004488 was not significantly reduced (Figure 1(d)). We observed that the expression of circ_0004488 was higher in human cervical cancer than in adjacent normal tissues, which correlated to the high levels of circ_0004488 in cervical cancer cells (Figure 1(e)).

We used qRT-PCR to determine whether the expression of circ_0004488 differed between paclitaxel-resistant SiHa (SiHa/PTX) cells and SiHa cells, given that CSCs are known to play a role in drug resistance in cancer cells [1]. Circ_0004488 expression was observed to be higher in SiHa/PTX cells than in SiHa cells (Figure 1(f)). In addition, qRT-PCR was used to evaluate the expression of circ_0004488 in PTX-sensitive and PTX-resistant cervical cancer tissue samples. Circ_0004488 expression was discovered to be higher in PTX-resistant CC tissues compared to PTX-sensitive CC tissues (Figure 1(g)).

The Kaplan–Meier survival curves showed that patients with cervical cancer who had greater levels of circ_0004488 had a worse overall survival rate (Figure 1(h)). In addition, we used qRT-PCR to determine the cellular location of circ_0004488 and found that it was mostly localized in the cytoplasm of cervical cancer cells (Figure 1(i)). Based on these results, we hypothesize that circ_0004488 is an oncogene and may contribute to paclitaxel resistance in cervical cancer.

### 3.2. Circ_0004488 Knockdown Decreases Cervical Cancer Cell Invasion, Sphere Formation, and Paclitaxel Resistance

**Figure 2:**
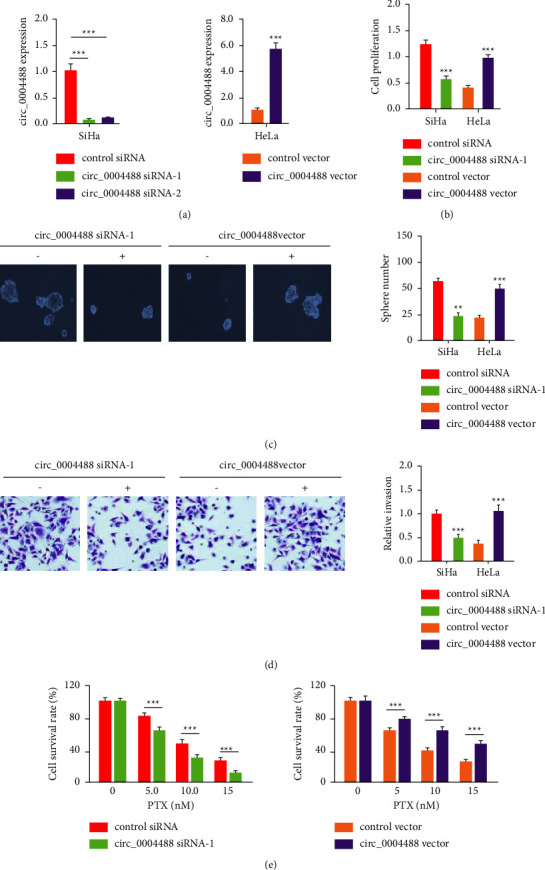
Circ_0004488 knockdown decreases cervical cancer cell invasion, sphere formation, and paclitaxel resistance. (a-b) Analysis of circ_0004488 by qRT-PCRs in (a) SiHa cells transfected with circ_0004488 siRNA or control siRNA and (b) HeLa cells transfected with the circ_0004488 vector or control vector. (b-e) After circ_0004488 was knocked down or overexpressed, CCK-8, sphere formation, transwell invasion, and cell viability tests were used to evaluate cervical cell proliferation (b), sphere formation (c), invasion (d), and cell viability (e). ^*∗∗∗*^*P* < 0.001.

To validate our hypothesis, siRNAs targeting circ_0004488 were transfected into SiHa cells, while a circ_0004488 expression vector was used to overexpress circ_0004488 in HeLa cells. Our qRT-PCR experiments confirmed whether circ_0004488 was downregulated or upregulated in cervical cancer cells (Figure 2(a)). Proliferation, invasion, and sphere formation were all inhibited in cervical cancer cells after circ_0004488 knockdown but were increased after circ_0004488 overexpression, which were relative to control cells, as determined by CCK-8, cell invasion, and sphere formation assays (Figures 2(b)–2(d)). Cell viability studies indicated that circ_0004488 knockdown increased the susceptibility of SiHa cells to paclitaxel relative to control cells (Figure 2(e)). However, when circ_0004488-overexpressing HeLa cells were compared to control cells, paclitaxel resistance was significantly increased (Figure 2(e)). These results demonstrate that elevated expression of circ_0004488 increases the growth, invasion, sphere formation, and paclitaxel resistance of cervical cancer cells.

### 3.3. Circ_0004488 Binds to miR-136 and Acts as a miR-136 Sponge

**Figure 3:**
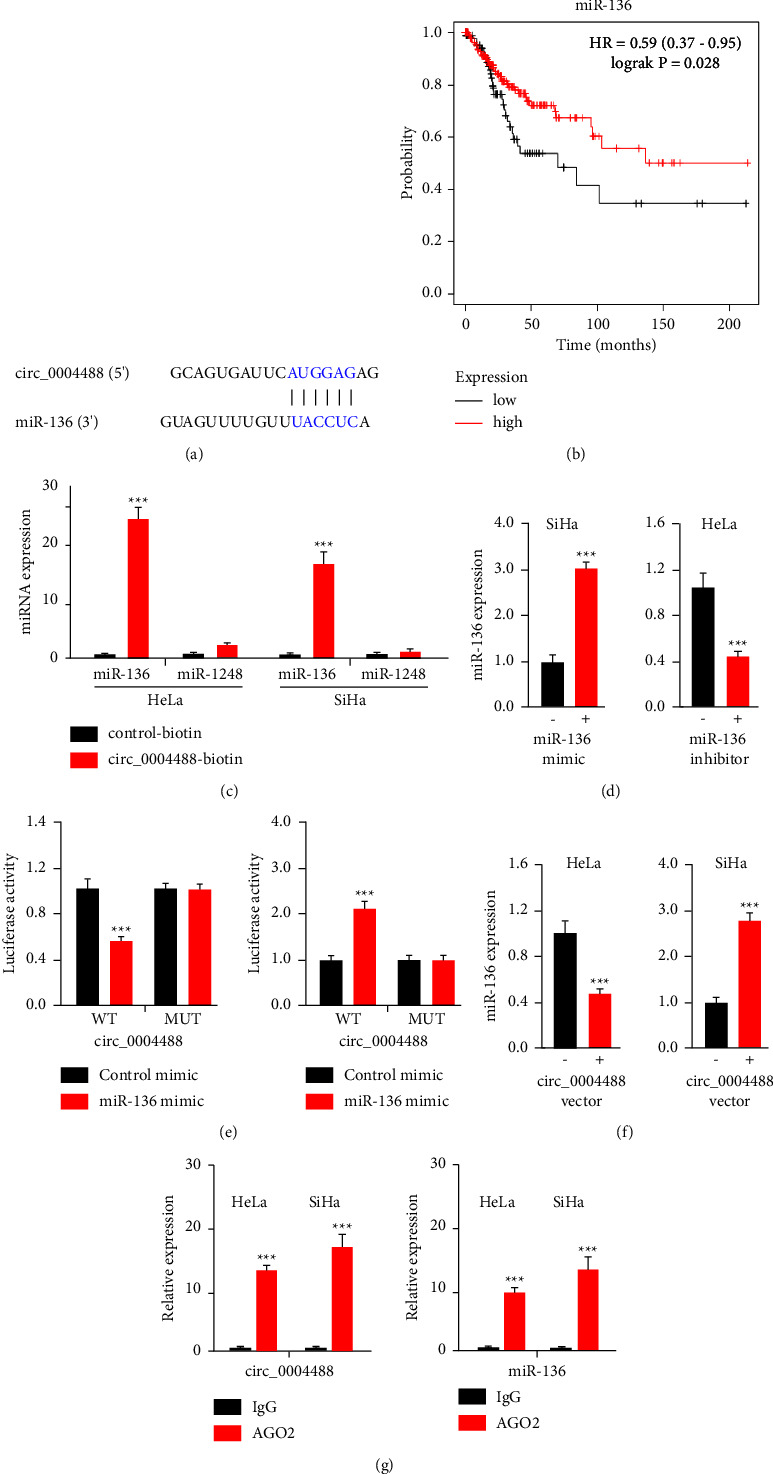
Circ_0004488 binds to miR-136 and acts as a molecular sponge for miR-136. (a) Circ_0004488 and miR-136 were found to potentially interact in the CircInteractome database. (b) The difference in survival rates between patients with high and low miR-136 expression in cervical cancer. (c) The circ_0004488 biotinylated probe and a control probe were transfected into cervical cancer cells. Circ_0004488 expression was quantified by qRT-PCR after streptavidin capture. As a negative control, we employed miR-1248. (d) MiR-136 expression in miR-136 mimic or inhibitor transfected cervical cancer cells. (e) Cervical cancer cells were transfected with miR-136 mimics (or miR-136 inhibitor) and either wild-type (WT) or mutant (MUT) circ_0004488 luciferase reporter vectors, and the relative luciferase activity was evaluated. (f) qRT-PCR experiments were used to analyze miR-136 expression in cervical cancer cells treated with a circ_0004488 vector or circ_0004488 siRNA. (g) In order to determine circ_0004488 and miR-136 expression in cervical cancer cells, the Ago2 RIP assay was performed. A qRT-PCR test was used to analyze circ_0004488 and miR-136 expression. ^*∗∗∗*^*P* < 0.001.

Given that circRNAs have been shown to operate as miRNA sponges and that circ_0004488 is mostly found in the cytoplasm of SiHa and HeLa cells, we speculated that circ_0004488 promotes cervical cancer chemoresistance by sponging miRNAs. In order to do this, the CircInteractome database was searched for miRNAs that may bind to circ_0004488. As a consequence, our bioinformatics prediction found a large number of miRNAs (Figure 3(a)). In the following investigations, we chose miR-136 since it has been shown to reduce cervical cancer cell proliferation, invasion, migration, and radiation resistance [11–13], as well as increase ovarian cancer cell sensitivity to paclitaxel [14, 15]. We discovered that miR-136 was a favorable predictive biomarker for cervical cancer patients using the Kaplan–Meier plotter database (Figure 3(b)).

We then investigated whether circ_0004488 could bind to miR-136 directly. Circ_0004488 was detected in cervical cancer cell lines using a biotin-labeled probe. Following the circ_0004488 pull-down, RNA was collected, and miR-136 levels were evaluated using qRT-PCR tests. Biotin-labeled circ_0004488 collected much more miR-136 than the control probe, as demonstrated in Figure 3(c). We cotransfected cervical cancer cells with miR-136 mimics or an inhibitor of miR-136 to investigate the interaction between miR-136 and circ_0004488 using a circ_0004488 luciferase vector containing the miR-136-binding site (Figure 3(d)). The luciferase activity of circ_0004488 was drastically suppressed by miR-136 mimics, in comparison to the control mimic (Figure 3(e)). Our luciferase reporter experiments further revealed that miR-136 inhibitor transfection dramatically increased circ_0004488-derived luciferase activity (Figure 3(e)). Transfection with miR-136 mimics or miR-136 inhibitors, on the other hand, did not affect the luciferase activity of MUT circ_0004488 (Figure 3(e)). The qRT-PCR experiments revealed that overexpression of circ_0004488 suppressed miR-136 expression, whereas downregulation of circ_0004488 increased miR-136 expression (Figure 3(f)). We discovered a substantial enrichment of endogenous circ_000448 and miR-136 in Ago2 immunoprecipitates using RNA immunoprecipitation techniques (Figure 3(g)). Circ_0004488 functions as a miR-136 sponge in SiHa and HeLa cells, according to these data.

### 3.4. MiR-136 Suppresses Cervical Cancer Cell Invasion, CSC-Like Features, and Paclitaxel Resistance

**Figure 4:**
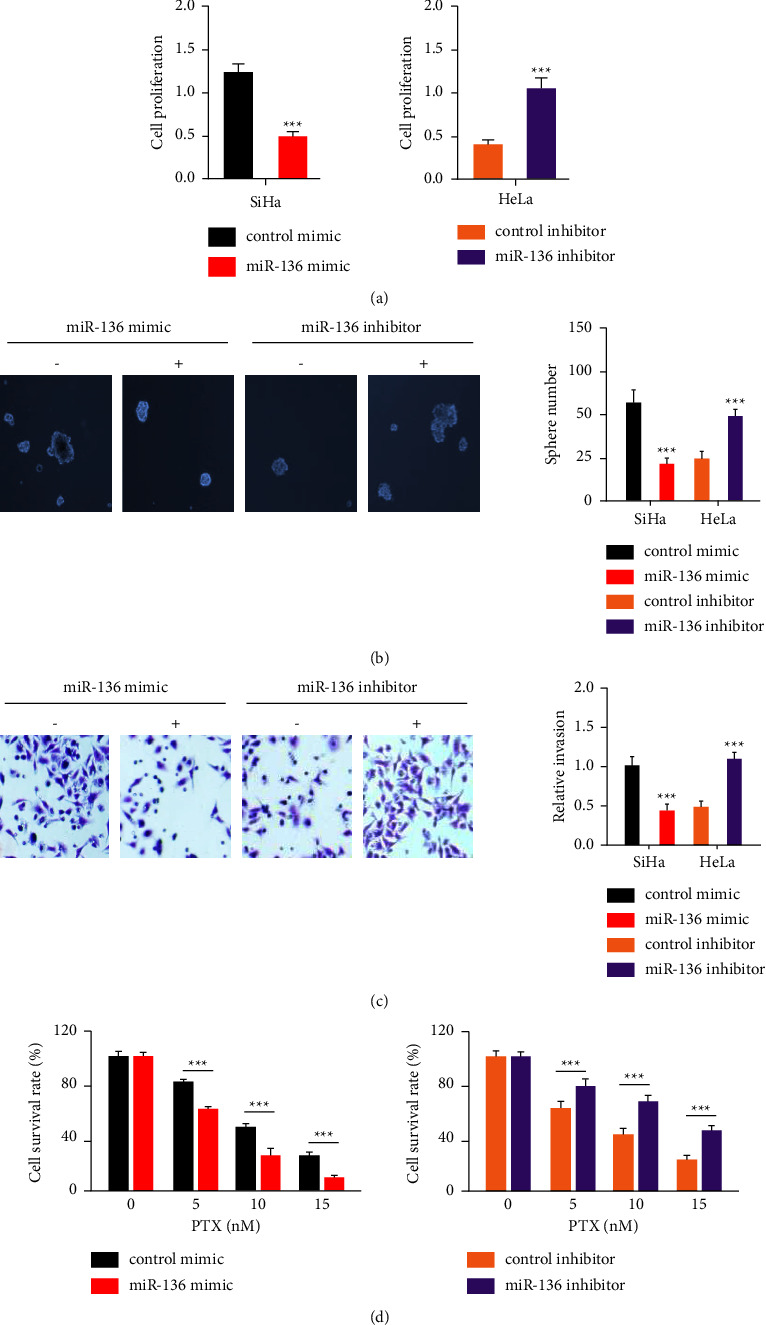
MiR-136 suppresses cervical cancer cell invasion, sphere formation, and paclitaxel resistance. (a–d) Overexpression or silencing of miR-136 was investigated for its effect on cervical cancer cell proliferation (a), sphere formation (b), invasion (c), and paclitaxel resistance (d). ^*∗∗∗*^*P* < 0.001.

We hypothesized that circ_0004488-induced reduction of miR-136 is a critical mechanism underpinning CSC-like phenotypes and paclitaxel resistance based on our findings. We overexpressed or shut down miR-136 in cervical cancer cells to study its involvement and then performed cell functional studies (Figure 4(a)). When miR-136 was overexpressed, it significantly reduced cervical cancer cell growth, invasion, and sphere formation (Figures 4(b) and 4(c)). However, miR-136-depleted cervical cancer cells were less able to grow, invade, and form spheres than control cells (Figures 4(b) and 4(c)). Experiments on cell viability also showed that SiHa cells were more sensitive to paclitaxel when miR-136 was overexpressed (Figure 4(d)). Our results also showed that paclitaxel resistance in HeLa cells was enhanced by the downregulation of miR-136 (Figure 4(d)). Our results also showed that paclitaxel resistance in HeLa cells was enhanced by the downregulation of miR-136 (Figure 4(d)). These results demonstrate that miR-136 inhibits tumor growth and enhances paclitaxel sensitivity in cervical cancer cells.

### 3.5. MiR-136 Directly Regulates MEX3C in Cervical Cancer Cells

**Figure 5:**
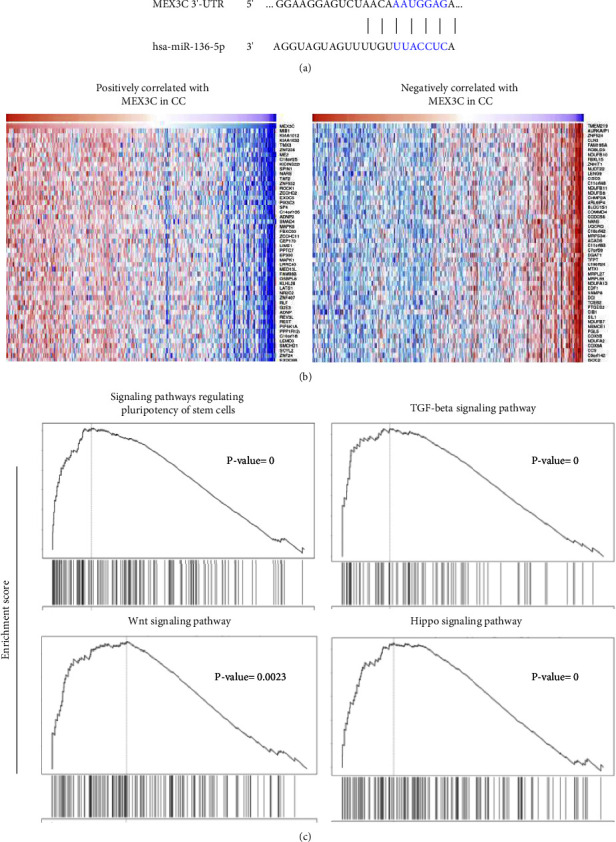
Coexpression analysis of MEX3C in cervical cancer tissues. (a) Sequence alignment between miR-136 and MEX3C (target scan database). (b) MEX3C coexpressed genes in TCGA cervical cancer tissues were investigated using the LinkedOmics database. The correlation of MEX3C with genes expressed in the cervical cancer cohort was studied using the Pearson test. Heat maps depicting genes in cervical cancer tissues that are positively (A) and negatively (B) linked to MEX3C. (c) GSEA revealed that elevated MEX3C expression was linked to CSC-related signaling pathways in cervical cancer tissues.

MiRNAs regulate the levels of their genes by binding to 3′-UTRs [10]. We utilized the TargetScan database and meta-analysis to discover miR-136 downstream genes (Figure 5(a)). Despite the fact that miR-136 is expected to have a wide number of downstream target genes, we chose MEX3C for our investigation since it has been linked to cancer development and chemoresistance stem cells [8, 9]. We analyzed MEX3C coexpressed genes in TCGA cervical cancer tissues using the LinkedOmics database.  Figure 5(b) depicts the genes that demonstrated a significant positive or negative connection with MEX3C.

To explore the functions of MEX3C in cervical cancer, MEX3C-related genes were then investigated using gene ontology (GO) and KEGG pathways. MEX3C, a coexpressed gene, may be implicated in the control of signaling pathways governing the pluripotency of stem cells, including TGF-beta, Wnt, and Hippo signaling pathways, according to GO and KEGG pathway analysis (Figure 5(c)). These findings showed that high MEX3C expression may be associated with CSC-like features and that circ_0004488 may affect sphere formation or paclitaxel sensitivity by raising MEX3C expression through sponging miR-136.

**Figure 6:**
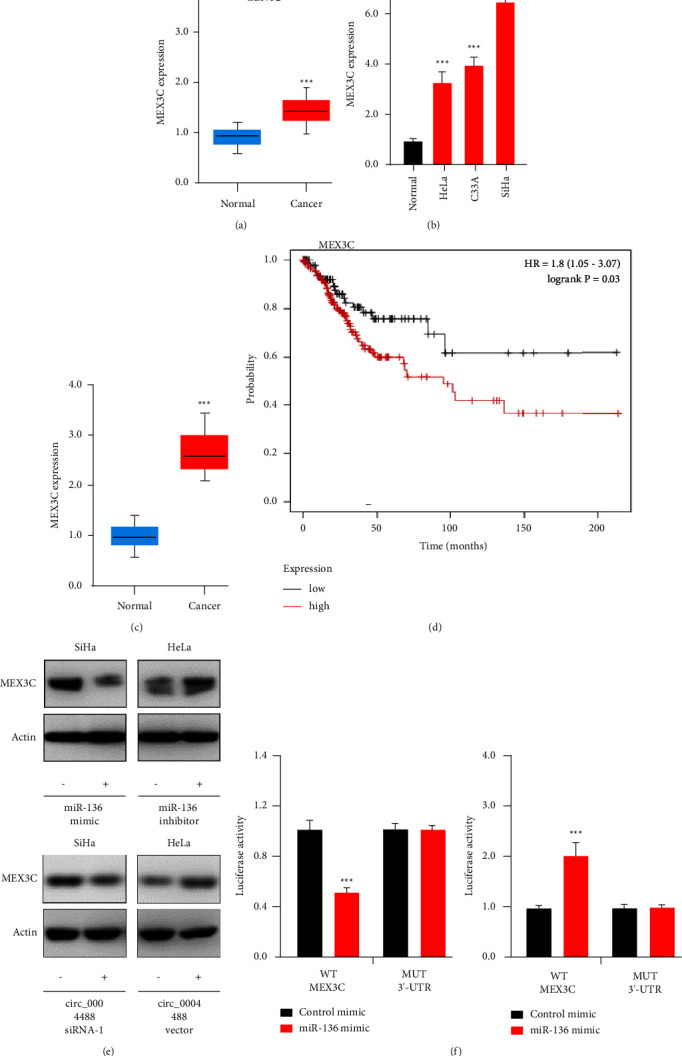
MiR-136 directly regulates MEX3C expression. (a) The expression of MEX3C in cervical cancer and normal tissues. (b) We used quantitative real-time polymerase chain reaction to examine MEX3C expression in cervical cancer cells and normal cells. (c) A comparison of MEX3C expression in cervical cancer and adjacent normal tissues. (d) The difference in survival rates between patients with high and low MEX3C expression in cervical cancer. (e) Western blotting demonstrates that downregulating MEX3C expression through knocking down circ_0004488 or overexpressing MiR-136. (f) After transfecting cervical cancer cells with miR-136 mimics (or miR-136 inhibitors) and wild-type (WT) or mutant (MUT) MEX3C 3′-UTR luciferase reporter vectors, the relative luciferase activity was determined. ^*∗∗∗*^*P* < 0.001.

To verify this hypothesis, we looked at MEX3C levels in the GENT database. Human cervical cancer tissues were shown to express much more MEX3C than normal tissues (Figure 6(a)). QRT-PCR results indicated that MEX3C expression was higher in cervical cancer cells than in normal cells (Figure 6(b)). MEX3C expression was shown to be significantly higher in cervical cancers compared to adjacent normal tissues (Figure 6(c)). Using the Kaplan–Meier plotter database, we found that patients with cervical cancer and high MEX3C expression had significantly worse overall survival (Figure 6(d)).

According to the findings of a western blotting study, when cervical cancer cell lines were transfected with circ_000448 siRNA or miR-136 mimics, the protein levels of MEX3C were lowered (Figure 6(e)). Conversely, circ_000448 overexpression or miR-136 knockdown can result in increased MEX3C protein expression (Figure 6(e)). Using a dual-luciferase reporter assay, we showed that in cervical cancer cells cotransfected with miR-136 mimics, the luciferase activity of the wild-type MEX3C 3′-UTR was much lower than that of control mimics (Figure 6(f)). Conversely, miR-136 inhibition increased the luciferase activity of the wild-type MEX3C 3′-UTR (Figure 6(f)). We discovered that miR-136 binding site alterations dramatically reversed the diminished luciferase activity produced by miR-136 mimic transfection (Figure 6(f)). Overall, our findings confirmed the notion that MEX3C was a direct target of miR-136.

### 3.6. MEX3C Overexpression Increases Cervical Cancer Cell Sphere Formation and Paclitaxel Resistance

**Figure 7:**
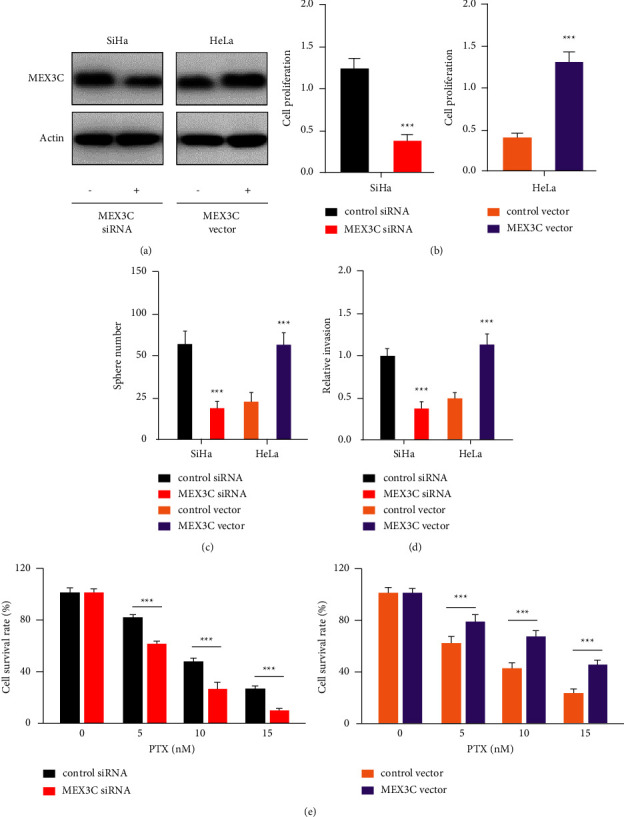
MEX3C overexpression increases cervical cancer cell sphere formation and paclitaxel resistance (a) examination of MEX3C in cervical cancer cells by western blotting after MEX3C overexpression or silencing. (b–e) The effects of MEX3C overexpression and silencing on cervical cancer cell proliferation (b), sphere formation (c), invasion (d), and paclitaxel resistance (e) were evaluated. ^*∗∗∗*^*P* < 0.001.

MEX3C behaves as an oncogene in colorectal cancer [20], bladder cancer [21], and osteosarcoma [22], but its biological relevance in cervical cancer is unclear. As a result, we utilized siRNA that targets MEX3C or a MEX3C expression vector to evaluate whether downregulation or upregulation of MEX3C could affect the proliferation, invasion, sphere formation, and paclitaxel resistance in cervical cancer cells. Our western blotting results show that cervical cancer cells significantly reduced or raised MEX3C expression (Figure 7(a)). Experiments using CCK-8, invasion, and sphere formation showed that MEX3C-knockdown cells had decreased cell proliferation, invasion, and sphere formation in comparison to control cells (Figures 7(b)–7(d)). By significantly increasing cell proliferation, invasion, and sphere formation, MEX3C overexpression aided in the progression of cervical cancer (Figures 7(b)–7(d)). By suppressing MEX3C expression, cervical cancer cells became more sensitive to paclitaxel (Figure 7(e)). The resistance to paclitaxel was promoted following overexpression of MEX3C (Figure 7(e)). These findings supported the idea that circ_000448 increases sphere formation or paclitaxel resistance through increasing MEX3C expression by affecting the antioncogenic activity of miR-136.

### 3.7. Circ_000448 Overexpression Stimulates Cervical Cancer Development

**Figure 8:**
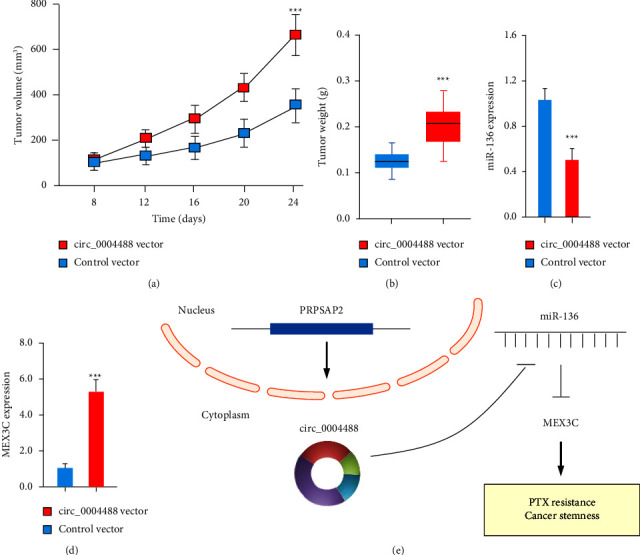
Circ_000448 overexpression stimulates cervical cancer development in vivo. (a) Nude mice were subcutaneously injected with an equivalent quantity of circ_000448-overexpressing HeLa cells or control cells. Tumor volumes (a) and weights (b) were measured. (c, d) In tumor xenograft experiments, the circ_000448 vector group had lower levels of miR-136 and higher levels of MEX3C than the control group. (e) The functions of the circ_000448/miR-136/MEX3C axis in cervical cancer chemoresistance are depicted in this schematic diagram. ^*∗∗∗*^*P* < 0.001.

To investigate circ_000448's role in cervical cancer formation in vivo, we stably transfected HeLa cells with an expression vector for circ_000448 or a control vector and implanted the cells subcutaneously into BALB/c nude mice. Tumor xenograft experiments showed that the circ_000448 vector group had much heavier and more numerous tumors compared to the control vector group (Figures 8(a) and 8(b)). Tumor tissues from the circ_000448 vector group showed a decrease in miR-136 and an increase in MEX3C after qRT-PCR analysis, in contrast to those from the control vector group (Figures 8(c) and 8(d)). Taken together, our results demonstrate that circ_000448 enhances CSC-like properties and paclitaxel resistance through the miR-136/MEX3C axis (Figure 8(e)). The development of circRNA-based therapies for cervical cancer may benefit from this signaling mechanism.

## 4. Discussion

Cervical cancer, a common gynecological malignancy, is a significant cause of mortality and morbidity among women worldwide [22]. The anticancer medicine PTX stabilizes microtubules and reduces their dynamicity, resulting in cell death and mitotic arrest [23]. One of the most efficient anticancer drugs for cervical cancer is PTX [24]. Increased chemoresistance to PTX definitely reduces the effectiveness of cervical cancer treatment [5]. CSCs are frequently drug-resistant, resulting in treatment failure, recurrence, and metastasis [1]. CircRNAs are known to have critical roles in regulating CSC self-renewal [25]. Paclitaxel-resistant processes, on the other hand, are complicated and poorly understood, and few studies on the underlying molecular process of CSC-associated circRNAs in cervical cancer have been done.

Our results show that circ_000448 is highly expressed in CSC-enriched populations of cervical cancer cell lines and is upregulated in paclitaxel-resistant cervical cancer cells. Furthermore, knocking down circ_000448 significantly reduced cell proliferation, invasion, sphere formation, and PTX resistance, as well as suppressing cervical cancer cell growth in vivo. When PTX-resistant tumor tissues were compared to PTX-sensitive tumor tissues, it was discovered that circ_000448 was increased in clinical samples from PTX-resistant patients. Thus, our results show that circ_000448 contributes to the maintenance of cervical cancer CSCs and that blocking circ_0004488 makes cervical cancer cells more sensitive to PTX. Our next study will need to use a larger sample size of clinical specimens to confirm these results.

Numerous circRNAs are expressed differently in cervical cancer cells than in normal cells, demonstrating that these circRNAs may have potential activities and biological importance [26]. CircRNAs represent a novel and promising method for cervical cancer diagnosis and treatment [26]. Circ_0000515 expression, for example, was much higher in cervical cancer tissues than in normal surrounding tissues [27]. The patients were then separated into two groups, with the mean expression of circ_0000515 serving as the cut-off value. Researchers found that cervical cancer patients with low circ_0000515 expression in their tumors had a significantly higher 5-year survival rate compared to those with high expression [27]. In addition, tumor stage, tumor size, and lymph node metastasis were all shown to be inversely correlated with circ_101996 expression levels in cervical cancer tissues [28].

Furthermore, higher circ_101996 levels have been connected to worse cervical cancer patient outcomes [28]. Circ_0043280 was shown to be downregulated in cervical cancer tissues as compared to normal cervical tissues [29]. In primary cervical cancer samples, lower circ_0043280 expression levels were shown to be related to prognostic clinical factors such as tumor size and lymph node metastasis [29]. In a multivariate Cox proportional hazards analysis, the expression levels of circ_0043280, tumor size, FIGO tumor stage, and lymph node metastasis were found to be independent predictors of cervical cancer [29]. Kaplan–Meier survival analysis [29] found that both overall survival and disease-free survival were poorer in individuals with low circ_0043280 expression. Our results confirmed the higher circ_0004488 levels previously reported in cervical cancer tissues [18]. Increased expression of circ_0004488 was related to a worse prognosis, as shown by our data. Based on these results, it is possible for circ_0004488 to act as a useful biomarker for the detection and evaluation of cervical cancer.

It has been shown that circRNA may function as miRNA sponges, influencing the expression of miRNA target genes in various human malignancies such as cervical cancer [30]. For instance, the invasiveness of cervical cancer is increased by circ_0000515 because it acts as a ceRNA for miR-326 [27]. In addition to its role as a sponge for miR-8075, circ_101996 has been demonstrated to promote the growth of cervical cancer cells and the invasion of bladder cancer cells [28]. Furthermore, circ_0043280 blocks miR-203a-3p [29], which slows the development and spread of cervical cancer tumors. Although circ_0004488 may affect gene expression by sponging miRNAs, its effect on cervical cancer chemoresistance remains unknown. In this study, we uncovered that circ_0004488 included a binding site for miR-136, and we were able to verify that circ_0004488 and miR-136 interact in cervical cancer cells. Our results were able to determine that circ_0004488 serves as miR-136's ceRNA in cervical cancer.

MiR-136 has been found to have a role in a wide variety of biological and clinical processes in recent years [11–15]. Bringing back normal levels of miR-136 expression has been associated with a decrease in peritoneal metastasis in gastric cancer [31]. When miR-136 was overexpressed, it inhibited epithelial-mesenchymal transition in head and neck cancer cells [32], whereas knocking it out increased EMT. By decreasing cell viability and boosting cell death and autophagy, overexpression of miR-136 enhanced cisplatin sensitivity in head and neck cancer cells [32]. Proliferation, invasion, migration, and resistance to radiation were all repressed by miR-136 in cervical cancer cells [11–13]. However, miR-136's role in PTX resistance in cervical cancer remains unclear. We found that miR-136 inhibited the development of cervical cancer cells by reducing their motility, invasion, proliferation, and sphere formation. The effectiveness of PTX is also enhanced when cervical cancer cells have been overexpressed with miR-136. Therefore, miR-136 might be a useful therapeutic target for the treatment of cervical cancer. Examining other miR-136 targets in cervical cancer is a priority for future research.

MicroRNAs (miRNAs) inhibit the levels of their targets by binding to and degrading certain mRNA targets [10]. This study demonstrated that miR-136 binds to the 3′-UTR of MEX3C mRNA, influencing energy metabolism, immune response, and cancer development. The activation of JNK signaling by MEX3C has been linked to bladder cancer development [8]. Ovarian cancer cells rely heavily on the JNK pathway to maintain resistance to PTX [9]. There has been much debate about MEX3C and its role in cervical CSCs and PTX resistance, but its specific function and mechanism remain unknown. We discovered that in vitro, overexpression of MEX3C promoted the invasion, proliferation, and sphere formation of cervical cancer cells. From these results, we concluded that circ_0004488 promotes CSC-like properties and PTX resistance by the modulation of the miR-136/MEX3C axis.

## 5. Conclusions

Circ_0004488 serves as an important miR-136 sponge, preventing the development of cervical cancer and PTX resistance via the miR-136/MEX3C axis.

## Data Availability

The data that support the findings of this study are available from the corresponding author upon reasonable request.

## References

[1] Visvader J. E., Lindeman G. J. (2012). Cancer stem cells: current status and evolving complexities. *Cell Stem Cell*.

[2] Robinson M., Gilbert S. F., Waters J. A. (2021). Characterization of SOX2, OCT4 and NANOG in ovarian cancer tumor-initiating cells. *Cancers*.

[3] Small W., Bacon M. A., Bajaj A. (2017). Cervical cancer: a global health crisis. *Cancer*.

[4] Laurie S. A., Ho A. L., Fury M. G., Sherman E., Pfister D. G. (2011). Systemic therapy in the management of metastatic or locally recurrent adenoid cystic carcinoma of the salivary glands: a systematic review. *The Lancet Oncology*.

[5] Markman M. (2014). Advances in cervical cancer pharmacotherapies. *Expert Review of Clinical Pharmacology*.

[6] Chhabra R. (2015). Cervical cancer stem cells: opportunities and challenges. *Journal of Cancer Research and Clinical Oncology*.

[7] Liu H., Wang H., Li C. (2016). Spheres from cervical cancer cells display stemness and cancer drug resistance. *Oncology Letters*.

[8] Chao H., Deng L., Xu F. (2019). <p>MEX3C regulates lipid metabolism to promote bladder tumorigenesis through JNK pathway</p&gt. *OncoTargets and Therapy*.

[9] Seino M., Okada M., Sakaki H. (2016). Time-staggered inhibition of JNK effectively sensitizes chemoresistant ovarian cancer cells to cisplatin and paclitaxel. *Oncology Reports*.

[10] Bartel D. P. (2004). MicroRNAs: genomics, biogenesis, mechanism, and function. *Cell*.

[11] Zhang J., Zhao X., Zhang J., Zheng X., Li F. (2018). Circular RNA hsa_circ_0023404 exerts an oncogenic role in cervical cancer through regulating miR-136/TFCP2/YAP pathway. *Biochemical and Biophysical Research Communications*.

[12] Zhao J., Yang T., Li L. (2020). <p>LncRNA FOXP4-AS1 is involved in cervical cancer progression via regulating miR-136-5p/CBX4 Axis</p&gt. *OncoTargets and Therapy*.

[13] Lu H. J., Jin P. Y., Tang Y. (2018). RETRACTED: microRNA-136 inhibits proliferation and promotes apoptosis and radiosensitivity of cervical carcinoma through the NF-*κ*B pathway by targeting E2F1. *Life Sciences*.

[14] Jeong J. Y., Kang H., Kim T. H. (2017). MicroRNA-136 inhibits cancer stem cell activity and enhances the anti-tumor effect of paclitaxel against chemoresistant ovarian cancer cells by targeting Notch3. *Cancer Letters*.

[15] Zhu J., Luo J. E., Chen Y., Wu Q. (2021). Circ_0061140 knockdown inhibits tumorigenesis and improves PTX sensitivity by regulating miR-136/CBX2 axis in ovarian cancer. *Journal of Ovarian Research*.

[16] Kristensen L. S., Andersen M. S., Stagsted L. V. W., Ebbesen K. K., Hansen T. B., Kjems J. (2019). The biogenesis, biology and characterization of circular RNAs. *Nature Reviews Genetics*.

[17] Shi Y., He R., Yang Y. (2020). Circular RNAs: novel biomarkers for cervical, ovarian and endometrial cancer (Review). *Oncology Reports*.

[18] Wang H., Zhao Y., Chen M., Cui J. (2017). Identification of novel long non-coding and circular RNAs in human papillomavirus-mediated cervical cancer. *Frontiers in Microbiology*.

[19] Lv Y., Cang W., Li Q. (2019). Erlotinib overcomes paclitaxel-resistant cancer stem cells by blocking the EGFR-CREB/GR*β*-IL-6 axis in MUC1-positive cervical cancer. *Oncogenesis*.

[20] Ruhl R., Rana S., Kelley K. (2018). microRNA-451a regulates colorectal cancer proliferation in response to radiation. *BMC Cancer*.

[21] Cao Y., Jiang F., Zhang S., Yang L., Sun Y. (2020). MEX3C promotes osteosarcoma malignant progression through negatively regulating FGF14. *J BUON*.

[22] Sung H., Ferlay J., Siegel R. L. (2021). Global cancer statistics 2020: GLOBOCAN estimates of incidence and mortality worldwide for 36 cancers in 185 countries. *CA: A Cancer Journal for Clinicians*.

[23] Xiao H., Verdier-Pinard P., Fernandez-Fuentes N. (2006). Insights into the mechanism of microtubule stabilization by Taxol. *Proceedings of the National Academy of Sciences of the United States of America*.

[24] Pectasides D., Kamposioras K., Papaxoinis G., Pectasides E. (2008). Chemotherapy for recurrent cervical cancer. *Cancer Treatment Reviews*.

[25] Lagunas-Rangel F. A. (2020). Circular RNAs and their participation in stemness of cancer. *Medical Oncology*.

[26] Chaichian S., Shafabakhsh R., Mirhashemi S. M., Moazzami B., Asemi Z. (2020). Circular RNAs: a novel biomarker for cervical cancer. *Journal of Cellular Physiology*.

[27] Tang Q., Chen Z., Zhao L., Xu H. (2019). Circular RNA hsa_circ_0000515 acts as a miR-326 sponge to promote cervical cancer progression through up-regulation of ELK1. *Aging (Albany NY)*.

[28] Song T., Xu A., Zhang Z. (2019). CircRNA hsa_circRNA_101996 increases cervical cancer proliferation and invasion through activating TPX2 expression by restraining miR-8075. *Journal of Cellular Physiology*.

[29] Zhang C., Liu P., Huang J. (2021). Circular RNA hsa_circ_0043280 inhibits cervical cancer tumor growth and metastasis via miR-203a-3p/PAQR3 axis. *Cell Death & Disease*.

[30] Cheng T., Huang S. (2021). Roles of non-coding RNAs in cervical cancer metastasis. *Frontiers in Oncology*.

[31] Zheng J., Ge P., Liu X., Wei J., Wu G., Li X. (2017). MiR-136 inhibits gastric cancer-specific peritoneal metastasis by targeting HOXC10. *Tumour Biol*.

[32] Yang B., Zang J., Yuan W., Jiang X., Zhang F. (2021). The miR-136-5p/ROCK1 axis suppresses invasion and migration, and enhances cisplatin sensitivity in head and neck cancer cells. *Experimental and Therapeutic Medicine*.

